# Multimodal analysis of the effects of dexamethasone on high-altitude cerebral oedema: protocol for a pilot study

**DOI:** 10.1186/s13063-019-3681-0

**Published:** 2019-10-24

**Authors:** O. Fisher, R. A. Benson, S. Wayte, P. K. Kimani, C. Hutchinson, C. H. E. Imray

**Affiliations:** 1 University Hospital Coventry and Warwickshire, Clifford Bridge Road, Coventry, CV2 2DX UK; 2 University of Warwick, Coventry, UK; 3 University of Birmingham, Birmingham, UK

**Keywords:** Altitude, hypoxia, cerebral oedema, steroid, acute mountain sickness, MRI

## Abstract

**Background:**

Acute mountain sickness (AMS) is a cluster of symptoms that commonly occur in those ascending to high altitudes. Symptoms can include headaches, nausea, insomnia and fatigue. Exposure to high altitude can also lead to high-altitude cerebral oedema (HACE), which is a potential cause of death whilst mountaineering. Generally, AMS precedes the development of HACE. Historical studies have demonstrated the effectiveness of regular dexamethasone administration in reducing the symptoms of AMS. However, the mechanism by which dexamethasone works to reduce symptoms AMS remains poorly understood. Further studies, simulating altitude using hypoxic tents, have characterised the effect of prolonged exposure to normobaric hypoxia on cerebral oedema and blood flow using MRI. This randomised trial assesses the effect of dexamethasone on hypoxia-induced cerebral oedema in healthy adult volunteers.

**Methods/design:**

D4H is a double-blind placebo-controlled randomised trial assessing the effect of dexamethasone on hypoxia-induced cerebral oedema. In total, 20 volunteers were randomised in pairs to receive either 8.25 mg dexamethasone or normal saline placebo intravenously after 8 h of hypoxia with an FiO_2_ of 12%. Serial MRI images of the brain and spinal cord were obtained at hours 0, 7, 11, 22 and 26 of the study along with serum and urinary markers to correlate with the severity of cerebral oedema and the effect of the intervention.

**Discussion:**

MRI has been used to identify changes in cerebral vasculature in the development of AMS and HACE. Dexamethasone is effective at reducing the symptoms of AMS; however, the mechanism of this effect is unknown. If this study demonstrates a clear objective benefit of dexamethasone in this setting, future studies may be able to demonstrate that dexamethasone is an effective therapy for oedema associated with brain and spinal cord ischaemia beyond AMS.

**Trial registration:**

Clinicaltrials.gov, NCT03341676. Registered on 14 November 2017.

## Background

Acute mountain sickness (AMS) is a cluster of symptoms that commonly occur in those ascending to high altitudes (>1500 m), including headaches, nausea, insomnia and fatigue [1]. Exposure to high altitude can also lead to the more dangerous high-altitude cerebral oedema (HACE), which is a potential cause of death whilst mountaineering. Whether HACE and AMS belong to the same pathophysiological spectrum is a matter of debate, although generally AMS precedes the development of HACE.

Magnetic resonance imaging (MRI) studies have demonstrated the impact of prolonged periods at altitudes of up to 4350 m on cerebral white matter volume and cerebral blood flow. Hypoxia is associated with a compensatory increase in cerebral blood flow and a subsequent increase in brain volume, which can be demonstrated through MRI [2]. The increasing use of hypoxic tents to induce AMS has allowed interval imaging and a clearer demonstration of both the timing and patterns of oedema development in the brain [3]. MRI is useful for monitoring the brain during these periods, as directly measuring the intracranial pressure is difficult to do or justify in healthy volunteers whereas the intracranial pressure can be inferred indirectly using the change in the volume of different intracranial tissues [3]. Kallenberg et al. used diffusion-weighted imaging (DWI) to demonstrate that AMS sufferers have a decreased apparent diffusion coefficient (ADC), a measure of how freely water can move between tissues [4–6]. The drop in ADC suggests cytotoxic oedema is an important component of AMS. This mechanism has many similarities with the accepted pathophysiology of cytotoxic oedema during cerebral ischaemia [7]. Compared to healthy tissue, the ischaemic spinal cord, like the brain, also shows a drop in ADC on DWI accompanied by hyperintensity on T2-weighted imaging [8–11]. Zhang [11] used canine models to demonstrate that changes in DWI and T2-weighted imaging are visible within an hour of inducing spinal cord ischaemia.

Double-blind studies simulating altitude have already demonstrated the effectiveness of regular dexamethasone administration in reducing the symptoms of AMS. Johnson et al. compared 6 hourly dexamethasone (4 mg dose) to a placebo in 16 men exposed to a simulated altitude of 4570 m using a hypobaric chamber [12]. A meta-analysis by Tang et al. [13] of using oral dexamethasone to treat AMS calculated an odds ratio of 6.03 (95% confidence interval, 2.23–21.00, *p* ≥ 0.05) compared to a placebo.

Multiple peripheral serum biomarkers of cerebral or blood–brain barrier dysfunction are currently under investigation; however, none have previously been applied to AMS and HACE. Glial fibrillary acidic protein (GFAP) is an intermediate filament protein that is upregulated in astrocytes following neuronal injury. There is good experimental correlation with neurological damage after a stroke or traumatic brain injury. Purine nucleosides (adenosine, inosine and hypoxanthine) have also been found to be sensitive early markers of ischaemic brain events [14, 15]. α-1 acid glycoprotein has recently been shown to act as a more sensitive marker of proteinuria than the more widely utilised urinary protein, albumin [16]. In animal models, the presence of proteinuria has been demonstrated to precede cerebral oedema and has been demonstrated to modulate the permeability of the blood–brain barrier [17, 18], as well as correlating with exposure to hypoxia [19].

Despite evidence for its efficacy, the mechanism behind the effect of dexamethasone remains poorly understood. Therefore, it has not been validated as a true therapeutic solution to AMS or HACE. This study aims to provide evidence of the mechanisms of action and therapeutic potential of dexamethasone for the treatment of symptomatic AMS and other causes of cerebral and spinal cord oedema and to investigate and correlate changes in peripheral serum biomarkers in response to hypoxia with radiological findings.

## Methods/design

### Study design

D4H is a phase-I double-blind placebo-controlled randomised control trial assessing the effect of dexamethasone on hypoxia-induced cerebral oedema in healthy adult volunteers.

### Hypothesis

A single dose of intravenous dexamethasone given after 8 hours in normobaric hypoxia at 12% inspired oxygen concentration (FiO2) reduces the incidence or severity of the symptoms of AMS, as determined by MRI changes due to cerebral oedema.

### Primary objective

To identify any measurable differences in:

1. Lake Louise scores
2. prespecified MRI parameters

following prolonged normobaric hypoxia after administration of a single dose of intravenous dexamethasone compared to a placebo.

### Secondary objectives

1. To measure the integrity of the blood–brain barrier and astrocyte dysfunction using proxy measurements (serum purine levels and GFAP) and correlate these with primary outcome measures.
2. To correlate proteinuria with the primary outcome measures.

#### Sample size

Estimates from our previous observational study of MRI differences at normobaric hypoxia were used to determine the power [3]. It was assumed that dexamethasone will reverse outcome measures to their baseline values. Powers for pairwise comparisons at 22 h for three different significance levels (0.05, 0.10 and 0.20) and for three outcome measures that showed change at 22 h from baseline in the observational work were calculated by assuming that the data are normally distributed, (Table 1). Since most of the other outcomes showed smaller differences, it was decided to test hypotheses at 20% significance and make this study a pilot study.

**Table 1:** Power for different outcomes and significance levels

Outcome	Estimates from Sagoo et al. [3]		Power		
	Difference	Standard deviation	α = 0.05	α = 0.10	α = 0.20
Blood oxygen content, ml O_2_/dL blood	2.05	1.33	97	98	99
Whole-brain ADC × 10^–4^/mm^3^	0.5	0.45	78	86	93
Corpus callosum (genu) ADC × 10^–4^/mm^3^ (mean of right and left)	1.35	1.65	52	64	77

*ADC* apparent diffusion coefficient

#### Inclusion and exclusion criteria

Subjects between the ages of 18 and 35 with a body mass index <30 kg/m^2^ who have not been to an altitude greater than 1500 m within 12 weeks were invited to participate. They provided written informed consent, which was obtained by a medically qualified doctor, as delegated by the chief investigator.

Patients with any systemic illness, currently pregnant or breastfeeding, or with recorded contra-indications to taking dexamethasone or having an MRI were excluded. Patients with a medical history of hypertension, untreated respiratory disease, glaucoma, epilepsy, peptic ulcer, recent surgery or known intra-cranial pathology were also ineligible.

#### Randomisation

Once eligibility had been confirmed, participants were allocated to the treatment or placebo group via block randomisation. The participants were randomised in sex-matched pairs and the randomisation sequence allocated participants attending on the same day to the same study arm to prevent unintentional unblinding of participants or investigators during the visit. The randomisation list was generated by computer by a statistician. Except in the case of an adverse event (AE) requiring unblinding, only the statistician who generated the list and the pharmacist are not blinded to study allocation. They are not members of the trial management group.

#### Intervention

Subjects in the placebo group received a single dose of 0.9% sodium chloride (8.2 mL) as the placebo, which was administered as a bolus injection intravenously over 5 min at 8 h post-baseline (0 h). Subjects in the intervention group received a single dose of dexamethasone 8.25 mg (2.5 mL) made up to 8.2 mL with 0.9% sodium chloride, which also was administered as a bolus injection intravenously over 5 min at 8 h post-baseline (0 h).

#### Schedule of events

**Fig. 1:**
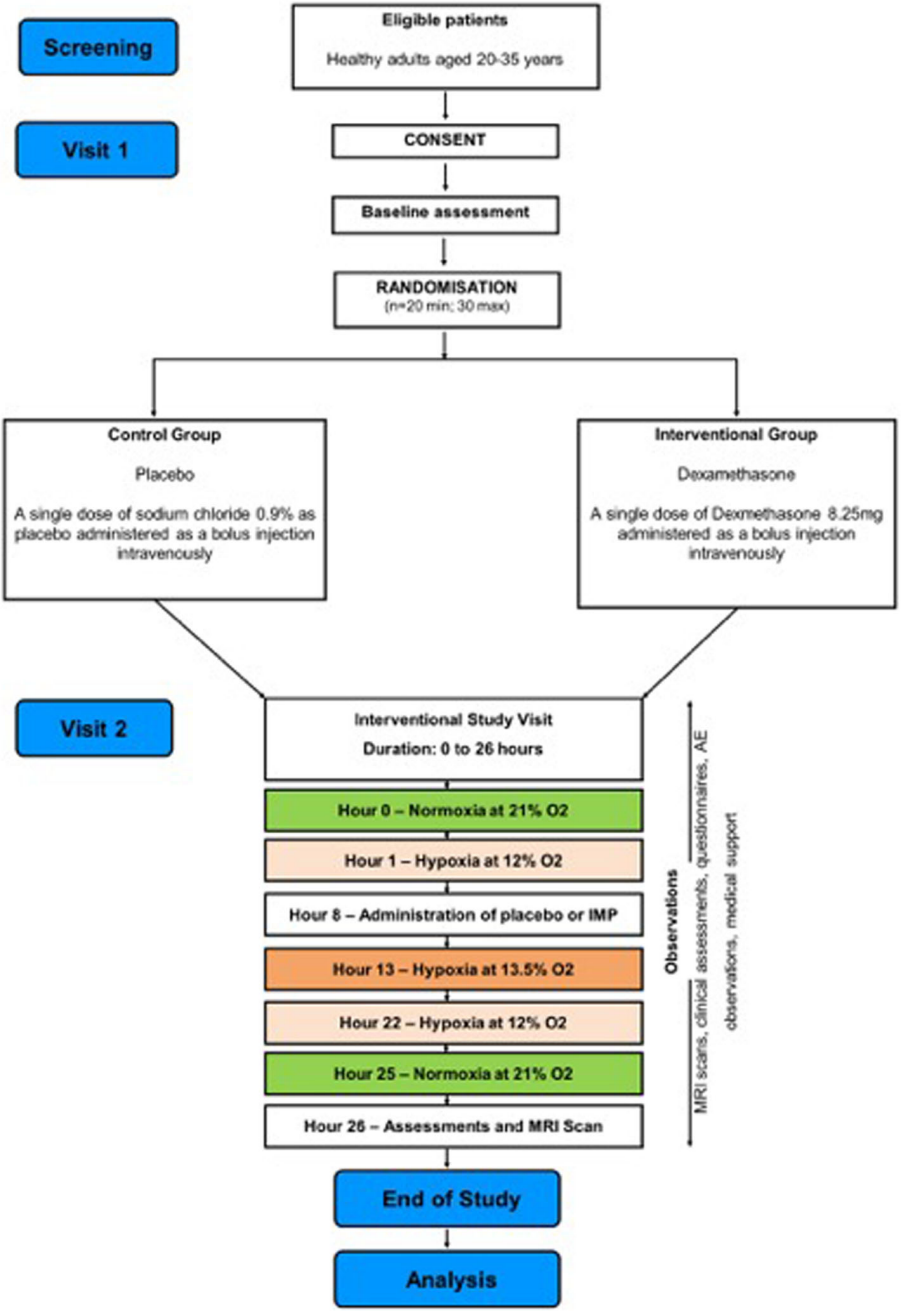
Participant flow diagram. IMP investigational medicinal product

Each subject visited the study site twice, the first time for a screening and consent visit and the second time for a period of 26 h for the study itself. A diagrammatic representation of patient flow through the trial is shown in Fig. 1. A full schedule of investigations, interventions and procedures to be performed at each study visit is shown in Table 2.

**Table 2:** Schedule of events

Observations and Assessments	Visit 1 – Screening	Randomisation	Visit 2 – Intervention (in hours)																											ET
			T = 0	1*	2	3	4	5	6	7	8	9	10	11	12	13	14	15	16	17	18	19	20	21	22	23	24	25	26	After T = 0
Eligibility confirmation	X																													
Informed consent	X																													
Medical history	X																													
Current medications	X																													
Demographics	X																													
MRI safety questionnaire	X		X																											
Vital signs	X		X		X		X		X		X			X											X		X		X	X
Blood test	X		X								X			X											X				X	
Urine Sample^***^	X		← Collected throughout the study →																											
Randomisation		X																												
Urine pregnancy test (females only)**			X																											
Venous cannulation			X																											
Finger-prick test			X								X			X											X				X	
MRI scan			X							X				X											X				X	
Lake Louise score			X		X		X		X		X			X											X					
Administration of IMP or placebo											X																			
Normoxia at 21% O_2_			X																									X	X	
Hypoxia at 12% O_2_				X	X	X	X	X	X	X	X	X	X	X	X										X	X	X			
Hypoxia at 13.5% O_2_																X	X	X	X	X	X	X	X	X						
Inspired O_2_ and CO_2_			X	← Every 15 mins →									← Every 30 mins →																	
AEs and SAEs			←Monitored throughout participation→																											X
End of study																													X	
Early termination																														X

*AE* adverse event, *ET* early termination, *IMP* investigational medicinal product, *SAE* serious adverse event

* Observations will be conducted +/- 30 mins of the stated hour wherever possible. Any deviations will be recorded on the protocol deviation log

**A urine pregnancy test must be carried out on all female participants at visit 2 (intervention visit) prior to conducting any study procedures

*** Urine will be collected in containers provided throughout the study duration, samples collected will be recorded for volume, prepared and stored at -80°C

#### Visit 1 – Screening

Volunteers were invited to attend University Hospitals Coventry and Warwickshire NHS Trust (UHCW) for screening and enrolment. Screening included baseline observations, a comprehensive health and MRI eligibility questionnaire, a pregnancy test if applicable and baseline blood tests. Consent included entering their personal information into the Health Research Authority’s Over-Volunteering Prevention System (TOPS). Once a potential participant had provided permission, the researcher sent a letter and copy of the participant information sheet to the participant’s general practitioner to inform them of their participation in the study.

#### Visit 2 – Intervention

##### Confirmation of eligibility and safety checks

The interventional study visit took place over 26 h in the MRI suite at UHCW. Any participants failing to meet the eligibility criteria on the day of intervention were withdrawn from the trial. Information on the last urine void time, a Lake Louise Acute Mountain Sickness self-assessment score, venous blood samples and baseline observations were recorded. The first MRI scan was then performed and used for baseline values.

##### Hypoxication

Following the assessment at hour 0, participants experienced 24 h of normobaric hypoxia at specific oxygen concentrations using a hypoxication tent (Everest Summit Hypoxic Generator, Hypoxic Systems, New York). The hypoxication tent is a self-contained structure measuring approximately 2 m (w) × 2.5 m (l) × 1.8 m (h), in which the oxygen levels are tightly controlled. Participants may leave the tent during the intervention, during which time they will wear a mask attached to a portable hypoxicator to make sure the inspired oxygen concentrations are controlled. Participants will continue with normal activities while in the tent, such as eating and sleeping. The time from which the participant begins to inspire hypoxicated air (FiO_2_ 12%) marks the start of hour 1. Subsequent observations were taken on the hour, plus or minus 30 min to allow for any unanticipated delays during the study visit. At hour 25, the participant returned to breathing oxygen at normal atmospheric pressure (FiO_2_ 21%). The hypoxication schedule is shown in Table 3.

**Table 3:** Schedule of participant hypoxication

Timeline	Inspired oxygen concentration (FiO_2_)
Baseline	Normal atmospheric O_2_ (21%)
Hours 1–12	12%
Hours 13–21	13.5%
Hours 22–24	12%
Hours 25–26	Normal atmospheric O_2_ (21%)

Three hypoxic generators were used to control the environment. Carbon dioxide build-up was controlled with an air pump (Bair Hugger™, 3M, Berkshire, UK). Air from the tent was pumped through two soda lime scrubbers (Spherasorb™, medical grade soda lime from Intersurgical Ltd, UK, in canisters by Draeger Medical UK Ltd). These were replaced after 12 h.

#### Outcome measures

##### MRI

Sequences performed in the brain have previously been found to produce significant results [3]. This imaging included assessment of the diameter and flow rate within the middle cerebral artery using 3D time of flight and 2D phase contrast MR angiography, respectively. In the present study, whole-brain DWI was used to produce ADC maps to quantify brain oedema. A T1-weighted total brain volume sequence was used to calculate changes in brain parenchymal and cerebrospinal fluid volumes as a result of cerebral oedema. T2*-based susceptibility-weighted imaging identified changes in the deep cerebral venous anatomy and found evidence of venous compression secondary to cerebral oedema. In addition, sequences were acquired to assess the cervical spinal cord volume and the total cord length. The flow rate in the internal carotid artery was measured using 2D phase-contrast MR angiography. The acquisition parameters used for all the MR sequences are given in Table 4.

**Table 4:** Technical MRI sequence parameters

Sequence	Purpose	TR (ms)	TE (ms)	Flip angle (°)	FoV (mm)	Matrix	Slice width/slice gap (mm)	Acquisition time	Comment
3D time of flight	Asses diameter MCA	27	5.7	20	210 × 210	384 × 256	1.2/0.0	3 min 10 s	
2D phase contrast	Assess flow in MCA	11	5.2	30	160 × 160	256 × 224	5.0	4 min 7 s	1 slice, 25 phases venc = 120 cm s^− 1^
Diffusion-weighted imaging	Produce ADC maps to quantify brain oedema	13,729	86.2	–	240 × 240	256 × 256	4.0/1.0	5 min 57 s	*b* = 0, 800 and 1000 s.mm^−2^
T1 3D FSPGR	Assess brain and CSF volume	8.5	3.3	12	240 × 180	256 × 256	1.8/0.0	5 min	TI = 400 ms Reconstructed to 0.9 mm slice width
SWI	Changes in venous volume	50.9	23.7	10	240 × 240	320 × 288	2.0/0.0	6 min 41 s	Reconstructed to 1 mm slice width
T2 3D FSE (cube)	Volume of the cervical spinal cord	1250	92.2	–	180 × 180	288 × 288	2.0/0.0	3 min 35 s	Reconstructed to 1 mm slice width
Sagittal T2	Assess total cord length	3406	106.3	–	440 × 440	384 × 288	4.0/0.0	1 min 56 s	Images combined to produce a single image
2D phase contrast	Assess flow in the internal carotid arteries	10.5	4.9	30	160 × 160	256 × 224	5.0	4 min 21 s	1 slice, 25 phases venc = 120 cm s^− 1^

*ADC* apparent diffusion coefficient, *CSF* cerebrospinal fluid, *FoV* field of view, *FSE* fast spin echo, *FSPGR* fast spoiled gradient echo, *MCA* middle cerebral artery, *SWI* susceptibility-weighted imaging, *TE* echo time, *TI* inversion time, *TR* repetition time

##### Blood sampling

Venous blood was taken immediately prior to each MRI scan and haematology and biochemistry markers were measured, including full blood count, renal function, erythrocyte sedimentation rate and levels of C-reactive protein. Serum samples were stored immediately at −80°C for a later GFAP analysis. Finger-prick capillary blood samples for purine assessment were also obtained at these time points.

##### Urine samples

Urine samples were collected and processed for every urine void during the intervention. The samples collected will be a spun down via centrifuge and were stored in a monitored freezer set to maintain temperatures of -70° to -85 °C. Samples were frozen within 1 hour of collection.

##### Clinical assessments

Vital signs were monitored every 2 h between hours 0–11 and hours 22–26. After the MRI scan at hour 0, the scans were repeated at hours 7, 11, 22 and 26. Participants were asked to complete the Lake Louise Acute Mountain Sickness Questionnaire at hours 2, 4, 6, 8, 11, 22 and 26. The Lake Louise score is a validated tool for diagnosing and scoring the severity of AMS [20]. Five questions (headache, gastrointestinal upset, fatigue, dizziness and sleep disturbance) were graded 0 (none) to 3 (severe) by participants. A total score of 3 is considered diagnostic of AMS. A score of 6–9 is considered moderate AMS and a score of 10 or above is considered severe AMS. In this study, the Lake Louise score was used to monitor participants for the development of AMS. If a participant had a score of 6 or above or an increase in total score of 2 or greater from the previous score, then consideration was made about withdrawing them from the study. Inspired O_2_ and CO_2_ were measured every 15 min from hours 1 to 9 and thereafter every 30 min from hours 10 to 24.

##### Preparation, storage and administration of the investigational medicinal product

The investigational medicinal product (IMP) was prepared for administration, labelled and stored in the trust’s aseptic laboratory for a maximum of 24 h prior to the study visit. Dexamethasone must not be stored above 25 °C and cannot be refrigerated or frozen. The study medication was stored in a temperature-controlled box. A temperature log ensured it remained within the acceptable temperature range until use. Any medication found to have breached the specified temperature range was discarded. On the intervention day, a member of the study team collected the prepared IMP for administration to the participants. Participants were randomised to receive a single dose of either the placebo or dexamethasone at hour 8 of the intervention visit. A medical doctor, as delegated by the chief investigator, prescribed and administered the study medication.

#### Statistical analysis

##### Summary of baseline data and flow of patients

Data exploration will include comparing the baseline characteristics of the volunteers in the placebo and dexamethasone groups. For continuous characteristics such as age, we will report the mean, median, range, interquartile range and standard deviation. Differences between the two groups will be compared using the Mann–Whitney *U* test. For categorical data such as gender, we will report the number and percentage of cases in each category. Fisher’s exact test will be used to compare the two groups.

##### Statistical analysis plan

As in Sagoo et al. [3], it thought plausible to assume that the outcomes would be normally distributed. We will assess this assumption using the residuals from models that assume the data are normally distributed. If the residuals are not normally distributed, we will transform the raw data and repeat the normality assumption check.

Ultimately the primary aim of the study is to compare outcomes between placebo and dexamethasone groups at 22 h. The comparisons could be made by performing *t*-tests using data collected at 22 h. This is, however, inefficient and so potentially to increase power, we will include outcome measures for all time points. To account for the correlation among measurements taken from the same volunteer at multiple time points, we will analyse data by fitting linear mixed models. Separate linear mixed models will be fitted for different outcomes. Each linear mixed model will include a fixed term(s) for the time at which a measurement was taken, a fixed term for the treatment group of the volunteer and an interaction term for time and treatment group. The interaction term enables us to make inference for the mean difference between the placebo and dexamethasone groups at different time points. If there is an imbalance of a baseline characteristic, a fixed term for the characteristic will be included in the model. Outcome measurement profiles over time will be assessed first. If the relationship is approximately linear, a linear term for time will be fitted. If not, time will be taken as a categorical variable.

For data taken at 22 h, we will report the estimated mean differences, the 95% confidence interval for the mean differences of the primary outcome measures and *p* values to test the hypothesis that the mean difference is zero. As mentioned previously, because this is a pilot study, the hypothesis will be tested at a 20% significance level. Also, for the same reason, we will not adjust for multiple hypotheses (corresponding to different outcome measures).

The above analysis plan will be used if it is plausible to assume that the data are normally distributed. If a transformation does not achieve normality, we will use non-parametric methods such as the Mann–Whitney U test.

#### Participant withdrawal criteria

A participant would be withdrawn from the study during the interventional visit in the following circumstances:

1. If the participant scores ‘severe’ for more than one symptom on the Lake Louise Mountain Sickness Score Questionnaire.
2. If it becomes medically necessary in the opinion of the clinician overseeing the study visit.
3. The participant requests to be withdrawn at any time.

In the event of withdrawal, the researcher will record the reason for discontinuation and any AEs in the participant’s notes.

#### Trial stopping rules

The trial will be stopped if:

1. A serious adverse reaction occurs that may be related to the administration of the IMP to a participant.
2. A severe non-serious adverse reaction occurs that may be related to the IMP, regardless of the system and organ class, to two participants.

In these circumstances, the trial steering committee, data-monitoring committee, sponsor, research ethics committee and the Medicines and Healthcare Products Regulatory Agency will be notified within 24 h of receipt of the initial report. Any participants actively involved in the trial will be withdrawn and all trial activities will be immediately discontinued.

#### Participant safety

A clinical member of the study staff was present in the study area at all times during the interventional study visit. Participants’ vital signs were monitored throughout the intervention period, except for hours 12–21, when it was expected that the participant would be sleeping, unless the participant became unwell. A thorough risk assessment of the study area was undertaken and approved. Unblinding of investigators and participants is permitted in the event of any AE, serious adverse event (SAE) or suspected unexpected serious adverse reaction (SUSAR). Unblinding will take place via codebreak envelopes stored within the MRI suite and pharmacy at UHCW.

#### Trial oversight

Trial oversight is provided by the trial management group. The trial steering committee will meet regularly to review progress and the safety of the study. There is also an independent data management group. The study sponsor, UHCW, is a National Institute for Health Research facility. The study was reviewed after the first two participants to ensure the safety and quality measures were met. After 20 patients had completed the protocol, a preliminary analysis was performed, and if sufficient differences had been seen, the study would have been closed to recruitment. If not, recruitment will continue to 30 patients. All potential AEs, as defined by good clinical practice (AEs, SAEs or SUSARs), were compiled by the trial manager and statistician and reviewed by the sponsor. All Grade IV AEs, all SAEs and all SUSARs were reported to the trial management group within 24 h. Participant information will be held securely at the trial sponsor site until study closeout, when they will be transferred to a third-party storage facility with appropriate security, and fire and flood provisions.

## Discussion

Previous studies have used MRI to identify changes in cerebral vasculature in the development of AMS and HACE. Since dexamethasone is therapeutic in this setting, the next stage of research is to demonstrate that dexamethasone administration has a direct effect on cerebral vasculature during a prolonged period of normobaric hypoxia to provide evidence of the mechanisms behind the development of AMS. If this study demonstrates a clear benefit of dexamethasone in this setting, the aim is to conduct further studies to include patients suffering from similar cerebral and spinal cord oedema due to other causes of cerebral hypoxia and spinal cord ischaemia. Ultimately, we would like to discover whether dexamethasone is an effective therapy for oedema associated with brain and spinal cord ischaemia.

### Trial status

This is protocol version 2.0, dated 25 July 2018. The scheduled start of recruitment was 2 February 2019. The anticipated end of recruitment was March 2020.

## Data Availability

The datasets generated and analysed during the current study will be available from the corresponding author or chief investigator on reasonable request.

## Abbreviations

ADC: Apparent diffusion coefficient
AE: Adverse event
AMS: Acute mountain sickness
DWI: Diffusion-weighted imaging
GFAP: Glial fibrillary acidic protein
HACE: High-altitude cerebral oedema
IMP: Investigational medicinal product
MRI: Magnetic resonance imaging
SAE: Serious adverse event
SUSAR: Suspected unexpected serious adverse reaction
SWI: Susceptibility-weighted imaging
UHCW: University Hospitals Coventry and Warwickshire NHS Trust
